# Supplementary material for: A Novel Dual Fluorochrome Near-Infrared Imaging Probe for Potential Alzheimer’s Enzyme Biomarkers-BACE1 and Cathepsin D
Source: Molecules. 2020 Jan 9;25(2):274. doi: 10.3390/molecules25020274 (PMC7024167; doi:10.3390/molecules25020274)
Supplement: Supplementary file 1 [file molecules-25-00274-s001.pdf]

Supplementary Material

A Novel Dual Fluorochrome Near-Infrared Imaging Probe for Potential Alzheimer's Enzyme Biomarkers-BACE1 and Cathepsin D

Jenny M. Tam [1], Lee Josephson [2], Alexander R. Pilozzi [3] and Xudong Huang [3,*]

[1] Wyss Institute and Harvard Medical School, Boston, MA 02115, USA; jenny.tam@wyss.harvard.edu
[2] Center for Molecular Imaging Research (CMIR), Department of Radiology, Massachusetts General Hospital and Harvard Medical School, Charlestown, MA 02129, USA; ljosephson@mgh.harvard.edu
[3] Neurochemistry Laboratory, Department of Psychiatry, Massachusetts General Hospital and Harvard Medical School, Charlestown, MA 02129, USA; APILOZZI@mgh.harvard.edu
* Correspondence: Huang.Xudong@mgh.harvard.edu; Tel: 617-724-9778; Fax: 617-726-4078

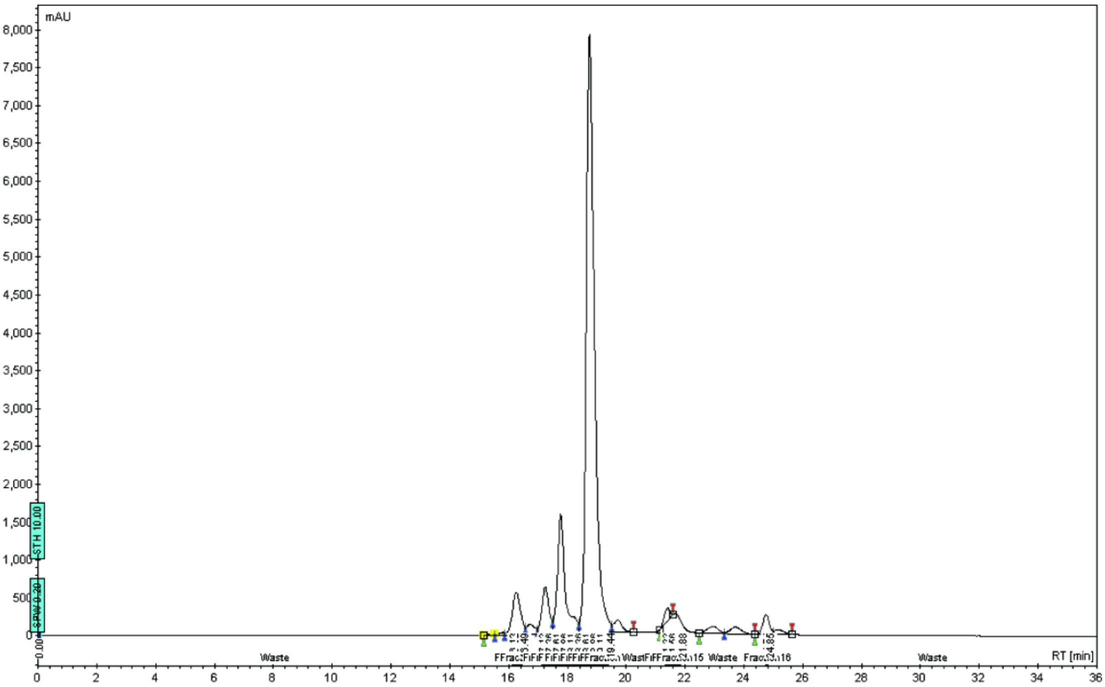

Supplementary Figure S1: HPLC chromatogram of labeled BACE1 peptide substrate.

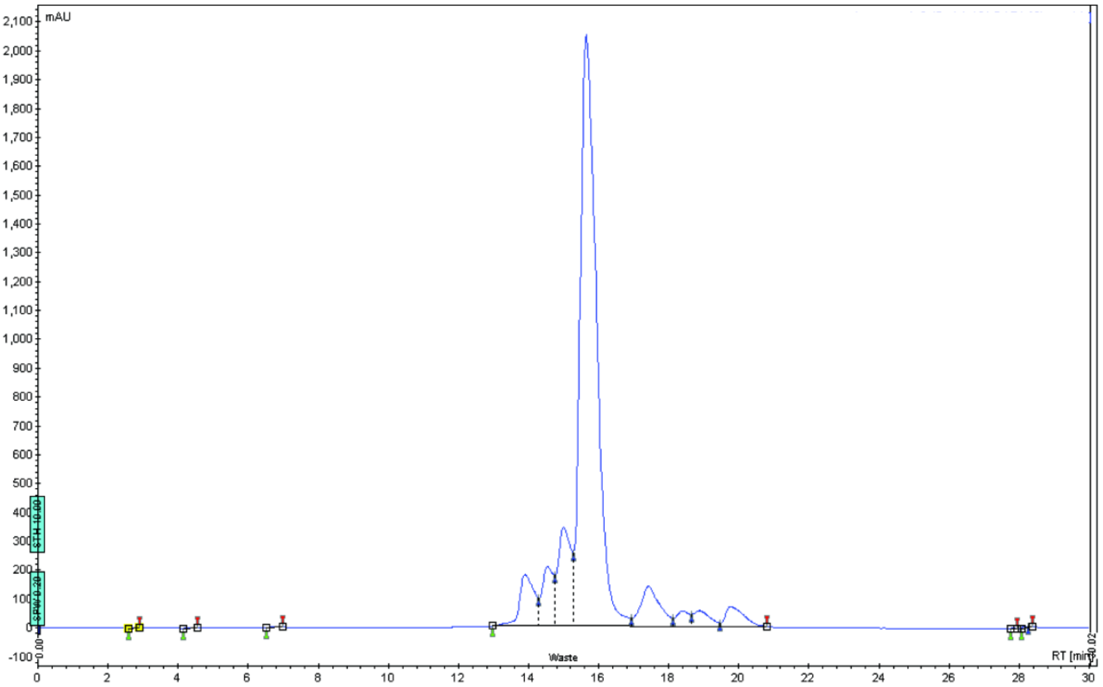

Supplementary Figure S2: HPLC chromatogram of labeled CatD peptide substrate.
